# Response Surface Methodology Applied to the Optimization of the Preparation of Antioxidant and Antidiabetic Extracts from *Phragmanthera capitata* (Spreng.) Balle: Effect of Particle Size, Powder-to-Solvent Ratio, and Temperature

**DOI:** 10.1155/2022/8397250

**Published:** 2022-09-01

**Authors:** Césaire Feudjio, Guy Sedar Singor Njateng, Mathilde Julie Klang, Muhammad Arfat Yameen, Muhammad Ahsan Khan, Jules-Roger Kuiate

**Affiliations:** ^1^Research Unit of Microbiology and Antimicrobial Substances, Faculty of Science, University of Dschang, P.O. Box 67, Dschang, Cameroon; ^2^COMSATS University of Information Technology, Abbottabad 22060, Pakistan

## Abstract

*Phragmanthera capitata* is a medicinal plant used in traditional medicine to treat several diseases, including diabetes. Its antioxidant properties and inhibitory effects on enzyme-carbohydrate digestion activities have been demonstrated. The present study aimed to provide data that can contribute to rationalizing the preparation of antioxidant and antidiabetic extracts from this plant. *P. capitata* (whole plant) growing on *Persea americana* was harvested at the fruiting stage. A response surface design-type central composite was applied to maximize the extraction yield, phenolic contents, and antioxidant and antidiabetic properties of the ethyl acetate extract of *P. capitata*. The influencing extraction factors were temperature, powder particle size, and solvent-to-powder ratio. The total phenolic content, total antioxidant capacity (TAC), DPPH scavenging ability, ferric reducing antioxidant power (FRAP), and antidiabetic (*α*-amylase and *α*-glucosidase inhibitory) effects of the extracts were determined using conventional methods. A temperature above 55°C contributed to the degradation of the extract, which was reflected in the GC-MS profile by a significant reduction in the number of compounds it contained. The optimal conditions were defined as 24.42°C for temperature, 250 *µ*m powder particle size, and 8.30 (v:w) solvent-to-powder ratio. This extraction protocol resulted in more than twice the extraction yield (3.05%), TTC (62.30 mg TAE/g), TAC (41.41 mg AAE/g), FRAP (186.56 mg AAE/g), and *α*-amylase (IC_50_ 15.05 *µ*g/mL) and *α*-glucosidase (IC_50_ 21.14 *µ*g/mL) inhibitory activities compared to our previous results. Additionally, these optimal conditions led experimentally to the extraction of higher phenolic content and to the attainment of higher antioxidant and antidiabetic activity, which closely matched the predicted values. Using these conditions, it is possible to prepare an antidiabetic phytomedicine from P. capitatathat can prevent oxidative stress complications. However, further complementary studies should be carried out considering other factors that influence the composition and pharmacological properties of the extract.

## 1. Introduction

Traditional medicine, particularly herbal medicine, plays an important role in primary healthcare systems in developing countries [1]. This is because of it easy access, it is generally well accepted by populations, has little or no toxic effects and can contribute to the economic growth of countries [2]. In some cases, it is the only alternative for rural populations. However, its practice remains mainly empirical and consequently presents significant limits [3]. Some of these limits include variation in chemical composition and pharmacological effects with ecological factors, plant intrinsic factors, and factors linked to the preparation of the phytomedicine itself. Indeed, herbal medicines are known to be complex mixtures comprising many constituents with qualitative and quantitative compositions depending on the batch of raw material and the methods of extract preparation (nature of the solvent, proportion, duration and temperature of extraction, etc.); it is almost impossible to obtain extracts of completely similar composition and therapeutic efficiency at any time [4]. In the light this, the success of phytomedicine usage requires, above all, a good level of technical and scientific mastery of standardization. Standardization is needed to ensure consistent pharmaceutical quality for herbal medicines by permitting standardized quality procedures at all stages of manufacturing, from raw materials to phytomedicine production [5].

Extraction is one of the main steps in the preparation of phytomedicines or in the discovery of potentially bioactive compounds from raw plant material. This process is influenced by many factors, such as the nature of the solvent, extraction temperature, time, powder-to-solvent ratio, size of the vegetable powder particles, and pH [6]. These factors influence not only the extraction yields but also the concentrations of the active ingredients and therefore the biological properties of the extract [7]. Optimization of the extraction is important, and the response surface methodology seems to be one of the best indicated. It allows us to explore the relationships between the dependent and independent parameters involved in an extraction process [6]. The first step consists of choosing the appropriate medicinal plant candidate.

*Phragmanthera capitata,* commonly called African mistletoe, is a woody semiparasitic shrub, belonging to the Loranthaceae family. It can grow on various hosts in secondary jungles, plantations, and bush savannah areas, from Sierra Leone to Western Cameroon, Fernando Po, extending across the Congo basin to RD Congo, Nigeria, Gabon, Ivory Coast, and Angola [8, 9]. Leaves, stems, or whole plants are widely used in traditional medicine to treat approximately 38 arrays of ailments [10, 11]. The most commonly used modes of preparation by healers are decoction, maceration, and infusion in water and palm wine [10, 12]. Some pharmacological studies carried out on various crude extracts and purified fractions have confirmed most biological properties of this plant against a number of ailments ranging from diabetes [12] to hypertension, epilepsy and sedation [13], infertility [14], obesity [15], bacterial and fungal infections [16], cancer, and inflammation [17]. Moreover, Ohikhena et al. [18] and Feudjio et al. [19] showed the antidiabetic activity of *P. capitata* through inhibition of *α* -amylase and *α*-glucosidase catalytic activity by ethyl acetate extract. Furthermore, the medicinal value of this plant is great due to its diversified phytochemical component content, including phenolics, flavonoids, alkaloids, and tannins [20] and its nontoxicity in experimental animals at appropriate doses [9]. This plant is therefore a suitable candidate for the formulation of antioxidant phytomedicine against oxidative stress diseases such as diabetes.

In a previous study, our team showed that the chemical composition and the antioxidant and antidiabetic properties of the extracts of this plant were influenced by the host plant, phenological stage, and solvent used to prepare the extracts. This plant, harvested from *Persea americana* and *Psidium guajava* during fruiting, gave better pharmacological results when extracted with ethyl acetate. Thus, the present study is a continuation of that work with the objective of optimizing the preparation of extracts with high antioxidant and antidiabetic effects using surface response methodology.

## 2. Materials and Methods

### 2.1. Plant Material

*Phragmanthera capitata* growing on *Persea americana* was harvested at the fruiting stage at 9 am in April 2018 in Bamendou village, Menoua division in the West Region of Cameroon. Authentication of the plant and its host was performed at the Cameroon National Herbarium (HNC) in Yaoundé by comparison of our plant specimens with registered voucher specimens nos. 24673/SRF/CAM (SRF: Cameroon forest reserve society) for *P. capitata* and 57756/HNC for *P. americana*. The plant sample was cleaned and dried for 3 weeks in a ventilated room at ambient temperature (22 ± 2°C). The dried plant material was ground using an herbal grinding system successively fitted with mesh sieves 14, 25, and 60 to obtain powder particle sizes of 250, 707, and 1410 *μ*m.

### 2.2. Methods

#### 2.2.1. Study Design

A response surface design-type central composite (RSD-CC) was used to investigate and optimize the extraction yield, total phenolic content, total flavonoid and flavonol content, total tannin content, total antioxidant capacity, DPPH scavenging activity, ferric reducing antioxidant power (FRAP), and *α*-amylase and *α*-glucosidase enzymatic inhibitory activity. The controlled factors, at three different levels, selected based on the data reported in the literature and preliminary assays, included temperature (A, 22 ± 2°C/48 h, 55°C/15 min, and 70°C/15 min), powder particle size (B, 250 *µ*m, 700 *µ*m, and 1400 *µ*m), and solvent-to-powder ratio (C, 3 : 1, 5 : 1, and 10 : 1 (v:w)) (Table 1). The measured effects (responses) included extraction yield, TPC, TFC, TFnC, TTC, FRAP activity, DPPH scavenging activity, and *α*-amylase and *α*-glucosidase extract inhibitory effects. From the performed experimental design, 27 experiments were conducted (Table 2). The relationship between the responses and the factors was described and fitted into the following polynomial regression equation:

(1) $$\begin{array}{c} Y=a_{0}+b_{1}A+b_{2}B+b_{3}C+c_{12}AB+C_{13}AC+C_{23}BC+d_{1}A^{2}+d_{2}B^{2}+d_{3}C^{2}. \end{array}$$

In this equation, *Y* is the predicted response; *a*_0_ is the interception coefficient; *b*_1_, *b*_2_, and *b*_3_ are the linear terms; *c*_12_, *c*_13_, and *c*_23_ are the interaction terms; and *d*_1_, *d*_2_, and *d*_3_ represent the squared coefficients of the independent variables. The model was statistically significant when the coefficient of determination was close to 100% and its probability of nullity *α* was less than 5% [21].

#### 2.2.2. Preparation of Extracts

The corresponding powder particle size of the plant material (250, 700, or 1400 *µ*m) was mixed with the specified solvent volume-to-powder proportion (1 : 3, 1 : 5, or 1 : 10) for the extraction process. The suspension was kept in a process of dynamic steeping for a specified time and temperature according to the matrix design of Table 2. A stirring heater set at 600 rpm was used to keep the suspension in the conical flask equipped with a reflux system under the specified temperature. Each resulting mixture was vacuum-filtered using Whatman filter paper no. 1, and the filtrates were evaporated at 40°C under vacuum through a Buchi R-210 evaporator. The resulting extracts were then dried at 40°C in an oven for 24 hours to remove the residual solvent and stored at 4°C for further studies. The extraction yield was calculated based on the initial dried powder mass [22]. Each extraction assay was performed in triplicate.

#### 2.2.3. GC-MS Analysis of Extracts

GC-MS was performed for ethyl acetate extracts achieved under defined optimal extraction conditions. This analysis was performed on a Perkin Elmer Clarus 600 GC/MS N6520204-E apparatus. The Perkin Elmer Clarus 600 GC System was fitted with an Elite-5MS capillary column (30 m long, 0.25 mm inner diameter, 0.25 *µ*m film thickness, and highest temperature of 350°C) coupled to a Perkin Elmer Clarus 600C MS. Helium (99.99% purity) was used as the carrier gas at a constant flow of 1 mL/min. The oven temperature was programmed from 40°C (held for 2 min) to 280°C (held for 10 min) at a rate of 5°C/min. The crude extracts were solubilized in chloroform and filtered with a syringe filter (Corning, 0.45 *µ*m). A volume of 1 *µ*L of the filtered extracts was injected into the column at a split ratio of 1 : 20. The percentage composition of each extract was expressed as a percentage of the peak area. The mass spectra were acquired within 50–550 m/z. The chemical constituents of the extracts were identified using gas chromatography retention indexes and mass spectra matching those of the standards available in the National Institute of Standards and Technology (NIST) library 2014.

#### 2.2.4. Determination of Phenolic Content, Antioxidants, and Inhibitory Activities of *α*-Amylase and *α*-Glucosidase of the Extracts

The total phenol content (TPC), total flavonoid content (TFC), total flavonol content (TFnC), total tannin content (TFC), total antioxidant capacity (TAC), ferric reducing antioxidant power (FRAP), and 2,2-diphenyl-1-picrylhydrazyl radical scavenging ability (DPPH-RSA) of the extracts as well as the inhibition of *α*-amylase and *α*-glucosidase by these extracts were determined as previously described [19]. Each assay was carried out in triplicate.

#### 2.2.5. Statistical Analysis

Minitab 19.0 software was used to generate the test matrix, to optimize the extraction conditions of *P. capitata*, and to analyze the results of the response surface design-type central composite. The response surface and iso-response curves used to visualize the combined effects of two independent variables on responses were generated with SigmaPlot 12.0 software.

The adequacy of the model was determined by evaluating the lack of fit, the coefficient of determination (*R*^2^), the adjusted coefficient of determination (*R*^2^ adjusted), and the regression (*p* test values) obtained from the ANOVA. The optimal extraction conditions maximizing the phenolic content, antioxidant and antiglycation activity, and *α*-amylase and *α*-glucosidase inhibitory effects were selected using the desirability function approach in Minitab 19.0 software.

## 3. Results

### 3.1. Effects of Combined Extraction Parameters on Responses

The results of the responses for the 27 runs following the experimental design are shown in Tables 3 and 4. All combined independent variables (extraction temperature, powder particle size, and solvent-to-powder ratio) had a variable effect on the 10 responses (extraction yield, TPC, TFC, TFnC, TTC, DPPH-RSA, and *α*-amylase and *α*-glucosidase inhibitory activities). Among these, the highest extraction yield (3.08%), TPC (727.95 GAE/g), antioxidant activities (TAC of 42.188 mg AAE/g and FRAP value of 187.76 mg AAE/g), and *α*-amylase and *α*-glucosidase inhibitory activities (IC_50_ of 13.646 and 21.119 *µ*g/mL, respectively) were observed in experimental run 16 under extraction conditions of 22°C/30 min extraction temperature, 250 *µ*m powder particle size, and 5 : 1 mL/g solvent-to-powder ratio.

### 3.2. Optimum Conditions of Extract Preparation for Phenolic Content and Antidiabetic Properties

**Figure 1:**
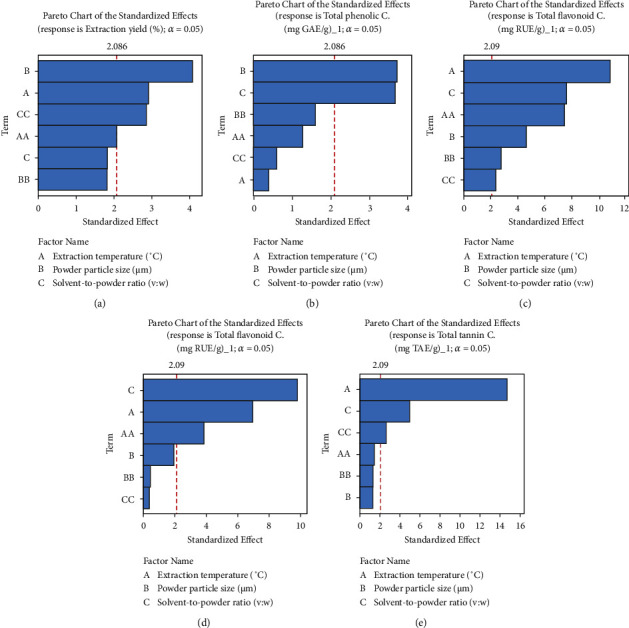
Pareto diagram highlighting the combined effects of temperature, powder particle size, and powder-to-solvent ratio on phenol content of ethyl acetate extract of *Phragmanthera capitata* growing at fruiting on *Persea americana*. AA: second-order effect of extraction temperature significantly affecting the extraction yield, TFC, and TFnC; BB: second-order effect of powder particle size significantly affecting the TTC; CC: second-order effect of solvent-to-powder ratio significantly affecting the extraction yield, TFC, and TTC.

**Figure 2:**
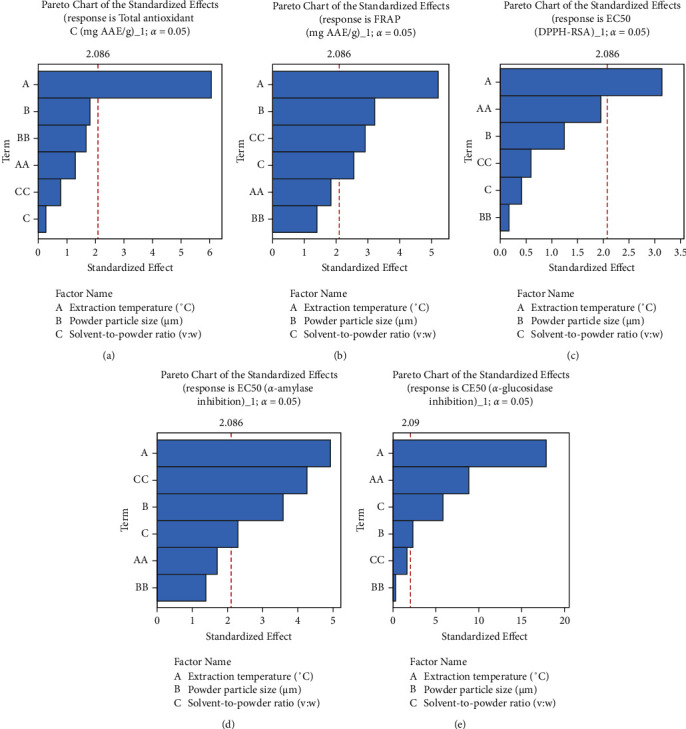
Pareto diagram highlighting the combined effects of temperature, powder particle size, and powder-to-solvent ratio on antioxidant activity and on inhibition of *α*-amylase and *α*-glucosidase enzymatic activities of ethyl acetate extract of *Phragmanthera capitata* growing at fruiting on *Persea americana*. AA: second-order effect of extraction temperature significantly affecting the liberation of *α*-glucosidase into the extract; BB: second-order effect of powder particle size not significant on the liberation of antioxidant and enzyme inhibitors into the extract; CC: second-order effect of solvent-to-powder ratio significantly affecting the liberation of ferric ion reducing antioxidant and *α*-amylase activities into the extract.

Pareto charts were used to identify factors (temperature (A), powder particle size (B), and powder-to-solvent ratio (C)) that significantly affected phenol content, antioxidant activity, and inhibitory activity exerted by the extract on carbohydrate digestion. These charts (Figures 1 and 2) show that the extraction conditions influenced the responses at different levels, but there were no valid interactions between them. The extraction yield, FRAP, and *α*-amylase inhibitory activity of the extracts were mostly influenced by powder particle size and extraction temperature (Figures 1(a), 2(b), and 2(d)). In contrast, the powder particle size and powder-to-solvent ratio had a significant effect on TPC (Figure 1(b)), while extraction temperature and solvent-to-powder ratio mostly influenced TFC, TFnC, TTC, and *α*-glucosidase inhibition (Figures 1(c)–1(e)). The extraction temperature significantly affected TAC and DPPH-RSA (Figures 2(a) and 2(c)). Furthermore, the second-order effect of extraction temperature (AA) produced a significant effect on the TFC, TFnC, and liberation of *α*-glucosidase inhibitors (Figures 1(c)–1(e)), while the quadratic effect of the powder-to-solvent ratio (CC) was found to produce a significant effect on the TFC, TTC, and liberation of ferric ion reducing antioxidants and *α*-amylase inhibitors (Figures 1(e), 2(b), and 2(d)).

From the multiple linear regression analysis of the 27 data entries, the relationships between the response and factors studied were second-order polynomial functions (Table 5). From these regression models, the extraction yield, TFC, TFnC, TTC, TAC, FRAP, *α*-amylase, and *α*-glucosidase effects were highly significant (*p* value = 0.000), with coefficients of determination (*R*^2^) ranging from 72.92 to 95.31. However, the regression models for data on the TPC and DPPH-RSA did not fit well, giving coefficients of determination *R*^2^ of 38.13 and 61.76, respectively.

**Figure 3:**
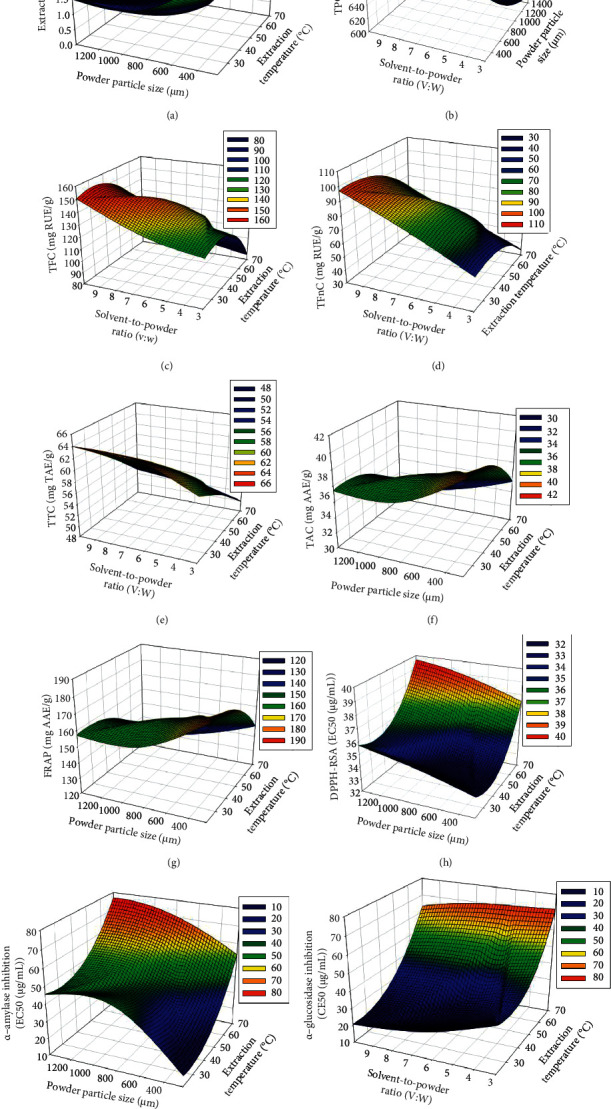
Response surface curves for the effect of two significant factors (extraction temperature, powder particle size, or solvent-to-powder ratio) on the responses (yield, phenolic content, and antioxidant and antidiabetic activities). Color gradients indicate the level of optimization (red: high, green: intermediate, and blue: low). (a) extraction yield, (b) total phenol content (TPC), (c) total flavonoid content (TFC), (d) total flavonol content (TFnC), (e) total tannin content (TTC), (f) total antioxidant content (TAC), (g) ferric reducing antioxidant power (FRAP), (h) DPPH-radical scavenging activity, (i) *α*-amylase inhibitory activity as IC_50_, and (j) *α*-glucosidase inhibitory activity as IC_50_.

Surface response plots illustrate the evolution of each response depending on the level of the most influencing factors (Figure 3). It was noted that the extraction yields increased when the powder particle size and extraction temperature decreased. Likewise, TAC, FRAP, DPPH-RSA, and *α*-amylase inhibition (low EC_50_) increased with decreasing extraction temperature and powder particle size (Figures 3(f)‒3(i)). TFC, TFnC, TTC, and *α*-glucosidase catalytic inhibition (low EC_50_) increased when the solvent-to-powder ratio increased at low extraction temperatures (Figures 3(c)–3(e) and 3(j)).

**Figure 4:**
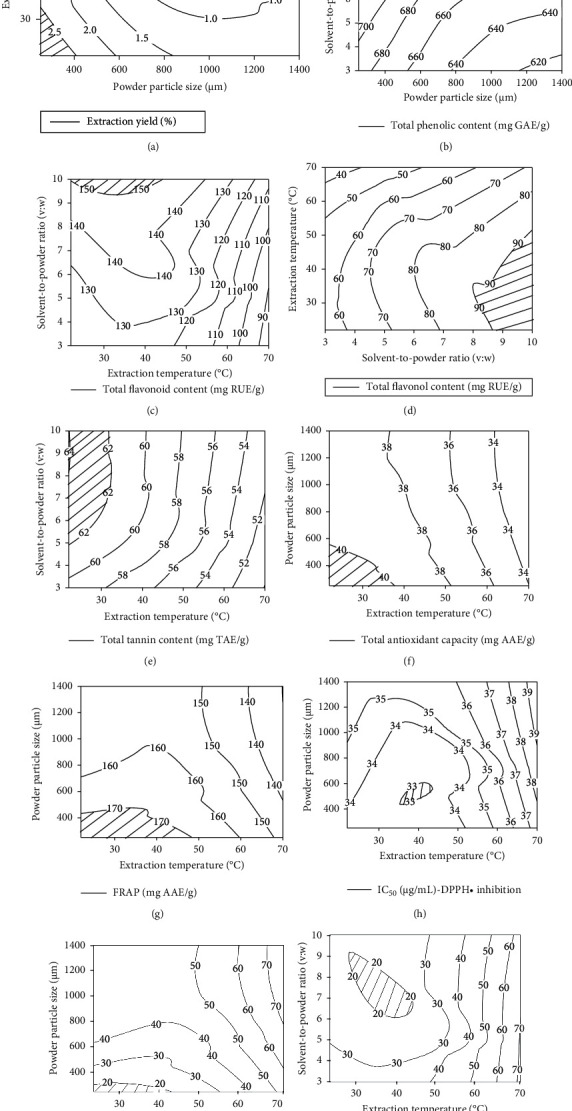
Iso-response curves of factors as a function of the most influencing independent variables. The hatched parts were the optimal areas. (a) Extraction yield (%); (b) total phenolic content (mg GAE/g); (c) total flavonoid content (mg RUE/g); (d) total flavonol content (mg RUE/g); (e) total tannin content (mg TAE/g); (f) total flavonoid content (mg RUE/g); (g) FRAP (mg AAE/g); (h) IC50 (*μ*g/mL), DPPH• inhibition; (i) IC50 (*μ*g/mL), amylase inhibition; (j) IC50 (*μ*g/mL), glucosidase inhibition.

Iso-response curves showed the optimal areas (hatched parts) corresponding to the maximal response (Figure 4). Thus, the extraction yields, TAC, FRAP, DPPH-RSA, and *α*-amylase inhibition (low EC_50_) were highest in the areas limited by extraction temperatures below 30°C and powder particle sizes less than 400 *µ*m (Figures 4(a) and 4(f)–4(i)). However, TFC, TFnC, TTC, and *α*-glucosidase catalytic inhibition (low EC_50_) were highest in the areas limited by a powder-to-solvent ratio of 8 : 1 and extraction temperatures below 30°C (Figures 4(c)–4(e) and 4(j)).

**Figure 5:**
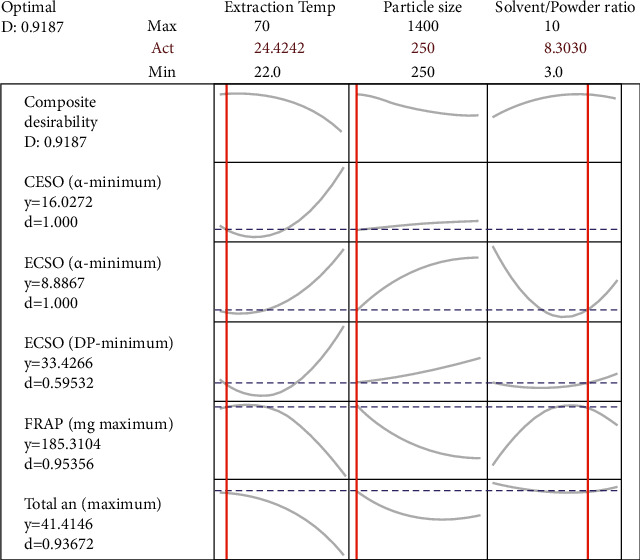
Prediction profile of optimal conditions for the extraction of antidiabetic and antioxidant principles from *P. capitata* harvested at fruiting on *Persea americana*.

The optimal extraction conditions for the yield, phenolic content, and antioxidant and antidiabetic activities of *P. capitata* using ethyl acetate as the extraction solvent were determined by superimposing their 3D response surfaces. The predicted optimum extraction conditions for maximum response (desirability close to 1) were obtained for 24.42°C temperature, 250 *µ*m powder particle size, and a 10 : 83 (g/mL) powder-to-solvent ratio (Figure 5).

The experimental responses obtained after running the assay under these optimal extraction conditions were compared to the predicted response values from the central composite experimental factorial design runs (Table 6). The experimental values obtained from the confirmation runs were within the predicted confidence interval values, thus proving the accuracy of the model.

### 3.3. GC-MS Compound Profile of the Ethyl Acetate Extracts of *P. capitata* Growing at Fruiting on *Persea americana*

**Figure 6:**
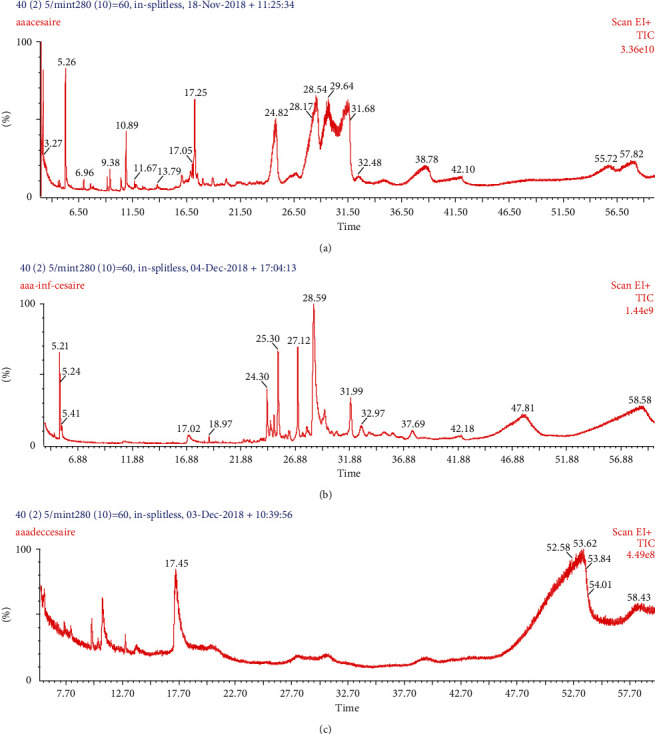
GC-MS chromatogram of *P. capitata* ethyl acetate extracts. (a) GC-MS profile of the optimized extract (extraction temperature of 24°C, powder particle size of 250 *µ*m, and powder-to-solvent ratio of 10 : 83 (V : W)). The numbers on the top of the peaks are the retention times of the compounds; for example, the major compound benzene, 1-(1,5-dimethyl-4-hexenyl)-4-methyl- was eluted at 28.54 min. (b) GC-MS chromatogram of the extract by applying an extraction temperature of 55°C, powder particle size of 250 *µ*m, and powder-to-solvent ratio of 10 : 83 (V : W). The major compound butan-2-one, 4-(3-hydroxy-2-methoxyphenyl)- was eluted at 28.59 min. (c) GC-MS chromatogram of the extract by applying an extraction temperature of 70°C, powder particle size of 250 *µ*m, and powder-to-solvent ratio of 10 : 83. The single compound oxirane, propyl was eluted at 17.45 min.

The GC-MS fingerprints were variable according to the extraction temperature (Figure 6). A total of 16 phytocompounds (Table 7) were identified in the *P. capitata*-optimized ethyl acetate extract (prepared using optimal extraction conditions: extraction temperature of 24°C, powder particle size of 250 *µ*m, and a powder-to-solvent ratio of 10 : 83 g/mL). The retention unit (ui) was used to characterize each compound instead of the retention time, which is variable as the analysis conditions. The major compounds were benzene, 1-(1,5-dimethyl-4-hexenyl)-4-methyl- (53.14%); 2-butanone, 4-(4-hydroxy-3-methoxyphenyl)- (29.91%); tetradecane (7.35%); hexadecane (3.21%); and octanal (2.10%). Seven compounds were identified in the extract by applying an extraction temperature of 55°C with an increase in the proportions of n-hexadecanoic acid (18.42%) and oxirane propyl (0.91%). Oxirane propyl (98.72%) was the only compound in the extract prepared at 70°C (Figure 6 and Table 8).

## 4. Discussion

Phenolics or polyphenols, including flavonoids, have received greater interest, as they have shown high antioxidant power and are correlated with many health benefits, including diabetes, cancer, and cardiovascular diseases [23]. Due to the chemical diversity and the many parameters that can influence the efficiency of antioxidant phenolic extractions from the plant, each phenolic source demands an individual approach for extraction and optimization [24]. Response surface design-type central composite (RSD-CC) is useful for the optimization of the extraction process and for studying the relationship between extraction parameters, phenolics, and correlated biological activities. In this study, the extraction temperature, powder particle size, and solvent-to-powder ratio were examined for their impact on the extraction yield, phenolic content, antioxidants, and carbohydrate digestive enzymes (measured effects or responses) of *P*. *capitata* extracts. From the RSD-CC used, all assessed extraction parameters showed a significant effect on all the responses without valid interaction between them.

The analysis of variance (ANOVA) for the measured effects (except total phenol content and DPPH-RSA) showed that the calculated and adjusted coefficients of determination (*R*^2^) were close to unity, ranging from 72.92% to 99.58%. This outcome indicates that the mathematical models of these plans were significant and that more than 72% or 99% of the response variables (extraction yield, TFC, TFnC, TTC, TAC, FRAP, and enzyme inhibitory activities) could be described by the RSD-CC model. In fact, according to Radojkovi et al. [25] and Azahar et al. [26], the closer the calculated and adjusted *R*^2^ values are to unity, the better and more significant the empirical model fits the actual data, and there is close agreement between the experimental results and the theoretical values predicted by the proposed model. Furthermore, the model *P* values (0.000–0.022) showed that the difference between the observed and predicted values was not more than 2.2%, bearing out the good accuracy of the developed model. Thus, this experimental design can be used to optimize the conditions under which *P. capitata* extracts should be prepared to obtain higher extraction yields, phenolic content, antioxidants, and antidiabetic activities.

Temperature was the main parameter that influenced the liberation of phenolics as well as antioxidants (through radical and redox reactions) and *α*-amylase and *α*-glucosidase inhibitors from *P. capitata*. There was a significant quadratic effect (*p* < 0.05) of temperature on TFC, TFnC, and *α*-glucosidase inhibitory activity, indicating both positive and negative effects on these phenolics and related activities. Increasing the extraction temperature from 22 to 40°C gave extracts with the highest phenolic content, while between 40 and 70°C, the amounts of phenolics, antioxidants, and inhibitors of enzymatic activities were significantly decreased. It is well known that a high extraction temperature softens the plant tissue and weakens phenol-polysaccharide and phenol-protein interactions, thus promoting the migration of molecules into the solvent and enhancing the extract efficiency related to them [27, 28]. This result was in accordance with a previous study. Roselló-Soto et al. [29] reported that the TPC and antioxidant activities of tiger nuts ethanol extracts increased from 25 to 37°C and then decreased. Altemimi et al. [30] concluded that the amounts of rutin and quercetin from peach and pumpkin extracts quickly increased and reached the maximum value with increasing extraction temperature from 30 to 41.08°C and then decreased due to thermal degradation of these flavonoids. Indeed, it was found that higher temperatures promoted degradation reactions such as the epimerization and oxidation of compounds, which could explain the loss of phenolic content and decrease the antioxidant and antidiabetic activities of extracts [31].

The effect of extraction temperature was reflected in the GC-MS profile of *P. capitata* ethyl acetate extracts at different extraction temperatures. In fact, GC-MS analysis identified 16 chemical markers in the *P. capitata* ethyl acetate extract at 25°C, including 2-butanone and 4-(4-hydroxy-3-methoxyphenyl); 7 compounds in the ethyl acetate extract at 55°C; and 1 compound in the extract achieved at 70°C. Rutin and quercetin were also previously identified in the HPLC profile of *P. capitata* ethyl acetate extract at 22°C [19]. These compounds could be linked to the broad spectrum of activity of *P. capitata*. Indeed, 2-butanone, 4-(4-hydroxy-3-methoxyphenyl)- is a polyphenolic alkanone, known antioxidant [32], and potential *α*-amylase and *α*-glucosidase inhibitor [33]. Rutin and quercetin are reference antioxidants [34] due to their redox properties, allowing them to scavenge free radicals, donate hydrogen atoms or electrons or chelate metal cations [35], and act as *α*-glucosidase and *α*-amylase inhibitors [36]. Butan-2-one, 4-(3-hydroxy-2-methoxyphenyl)- could be the product of the degradation reaction in the extract at 55°C, which, associated with the thermal degradation of rutin and quercetin, could contribute to the decrease in antioxidants and enzymatic inhibitory activities of the resulting extract. This result was also in accordance with a previous study that stated a decline in phenolic content and antioxidants from 55 to 65°C in *Funtumia elastica* [27].

After temperature, the particle size is another important parameter controlling the kinetics of mass transfer and the access of the solvent to soluble compounds [37]. In this study, increasing the extraction temperature from 22 to 30°C combined with the reduction in powder particle size below 400 *µ*m promoted the migration of antioxidants and *α*-amylase inhibitors into the extraction solution. This can be explained by the fact that the reduction in plant powder particle size leads to an increase in solubility and diffusion of bioactive components in the plant matrix due to the increase in the surface area available for mass transfer between the particle and the solvent [38, 39]. Makanjuola et al. [40] reported that the phenolic content and antioxidant properties in ground tea with particle sizes of 0.425 mm were higher than those in ground tea with particle sizes of 1.180 mm. A rapid increase in polysaccharide yield was observed when the particle size of *Codonopsis pilosula* powder decreased from 420 to 180 *μ*m, and the optimal particle size was 180 *μ*m; the maximum polysaccharide yield was 15.56% [41]. In contrast, catechin content and antioxidant properties in hydroalcoholic extracts of green tea decreased for particles of sizes less than 50 *μ*m, showing that the smallest particles can sediment and decrease the contact between particles and the extraction solvent, thereby reducing mass transfer efficiency [42]. Thus, the critical particle size should be defined for each plant matrix, hence the importance of optimization.

There was also a significant positive quadratic effect of the solvent-to-powder ratio on TFC, FRAP, and *α*-amylase inhibitory activity (*P* < 0.05), showing a positive effect on TFC. With extraction temperatures ranging from 22 to 30°C, the higher this ratio was, the higher the extracted TFC, antioxidant, and *α*-amylase inhibitors were. An increase in solvent volume was linked to more extraction efficiency, which could be due to the faster diffusion rate and better mass transfer leaching out of phenolic components from plant raw materials due to increases in the concentration gradient as a driving force [43–45]. The solvent-to-powder ratio beyond 5 : 1 mL/g was the most effective, in agreement with Predescu et al. [46], whose study reported that a solid-to-solvent ratio of 10 : 1 mL/g was the most effective for phenolic compound and antioxidant extraction from some plants. However, Andres et al. [47] reported a decrease in TPC in craft brewers' spent grain with increasing liquid-solid ratios, which took place until a 16 : 1 ratio, and a plateau was reached beyond that ratio. Montenegro-Landívar et al. [48] did not observe a significant effect on TPC when the solid/liquid ratio increased from 10 : 1 to 100 : 1 (v/w) when applying water extraction in orange and spinach wastes. Thus, Montenegro-Landívar et al. [48] concluded specifically that, apart from improving extraction yields, the knowledge of the optimal amount of solvent to use is of economic relevance.

The optimum extraction conditions were obtained for a desirability of 0.92 (close to 1). Prediction showed that an extraction temperature of 24.42°C/15 min, a powder particle size of 250 *µ*m, and an ethyl acetate-to-powder ratio of 8.30 : 1 mL/g can be considered the optimum extraction conditions maximizing the extraction yield, phenolic content, antioxidants, and *α*-amylase and *α*-glucosidase inhibitor extraction from *P*. *capitata*. Thus, this extraction protocol resulted in more than twice the extraction yield (3.05%), TTC (62.30 mg TAE/g), FRAP (186.56 mg AAE/g), and *α*-amylase (IC_50_ 15.05 *µ*g/mL) and *α*-glucosidase (IC_50_ 21.14 *µ*g/mL) inhibitory activities when compared to a previous study on *P. capitata* extraction, where only 1.45% extract yield, 15.31 mg TAE/g TTC, 66.17 mg AAE/g FRAP value, IC_50_ of 92.2 *µ*g/mL (*α*-amylase), and IC_50_ of 32.75 *µ*g/mL (*α*-glucosidase) were obtained using a temperature of 22°C/48 h, a powder particle size of 250 *µ*m, and ethyl acetate-to-powder of 5 : 1 mL/g [19].

Under the optimal conditions, the predicted values of all the responses were close to the experimental values, and all the experimental data were within ±95% prediction intervals, which confirms the accuracy and validation of the model. Therefore, it can be concluded that the model from RSD-CC was accurate and reliable enough for predicting the phenolic content, antioxidants (through radical and redox reactions), and inhibitors of *α*-amylase and *α*-glucosidase activities. Previously, the extraction parameters of bioactive compounds have been successfully optimized using the RSD-CC method, and some of them are in agreement with our optimized protocol to a lesser extent. Clodoveo et al. [49] found that the maximum polyphenol extraction efficiency from *Ceratonia siliqua* was achieved under the optimized factor combination of 35°C/15 min, 5 : 1 mL/g water-to-powder ratio, and 0.3 mm powder particle size. The standardized conditions for the extraction of polyphenols from pomegranate peel were found to be extraction room temperature of 28 to 30°C, solid-to-water ratio of 1 : 10, and particle size in the range of 100 to 400 *μ*m [50]. In contrast, the optimized conditions for nutraceuticals and antioxidant extraction from mulberry leaves were 70°C extraction temperature, extraction time of 40 min, water-to-leaf-powder ratio of 40 : 1 mL/g, and particle size of 25 *µ*m [43]. Abu Bakar et al. [6] determined a temperature of 79.07°C for 17.42 min with a solid-to-liquid ratio of 1 : 20 g/mL as the best extraction conditions for TPC, TFC, and xanthine oxidase inhibitory activity of *Euphorbia hirta*. Furthermore, an extraction temperature of 72.06°C and an ethanol-to-solvent ratio of 1 : 22 g/mL were the optimal conditions for the highest extraction yield and PTP-1B and *α*-glycosidase enzyme inhibition rates from Kursi Wufarikun Ziyabit [22]. These different results can be ascribed to the raw material studied, extraction methods and analyses, and the chemical nature of phenolic compounds in plant tissues.

## 5. Conclusion

The present study focused on certain extraction parameters to maximize the yield and biological properties of extracts obtained from *Phragmanthera capitata* whole plants. From the obtained results, this was achieved by applying the following extraction parameters: a temperature of 24.42°C, powder particle size of 250 *µ*m, and solvent-to-powder ratio of 8.30 mL/g. These conditions led to a higher yield and higher phenolic content of the extract and to better antioxidant and antidiabetic activities. Furthermore, the identified compounds in the ethyl acetate extract could be used as standardization markers of a possible phytomedicine from this plant. This needs to be confirmed through further studies.1

## Figures and Tables

**Table 1:** Combination scheme of factor (independent variable) levels used for optimization of the extraction process.

Factors	Code units		Variable levels	
Extraction temperature (°C)	A	22	55	70
Powder particle size (*μ*m)	B	250	707	1410
Solvent-to-powder ratio (v:w)	C	(3) 3 : 1	(5) 5 : 1	(10) 10 : 1

**Table 2:** The central composite experimental design for the extraction process.

Run order	Independent variables		
	Extraction temperature (°C)	Powder particle sizes (*μ*m)	Solvent/powder (v:w)
1	22	707	3
2	22	707	3
3	22	707	3
4	22	707	5
5	22	707	5
6	22	707	5
7	22	707	10
8	22	707	10
9	22	707	10
10	22	1400	5
11	22	1400	5
12	22	1400	5
13	22	707	5
14	22	707	5
15	22	707	5
16	22	250	5
17	22	250	5
18	22	250	5
19	22	707	5
20	22	707	5
21	22	707	5
22	55	707	5
23	55	707	5
24	55	707	5
25	70	707	5
26	70	707	5
27	70	707	5

**Table 3:** Response surface central composite design and results for extraction yield, total phenolic content, total flavonoid content, total flavonol content, and total tannin content.

No.	Independent variables			Response variables and their values				
	Extraction temperature (°C)	Powder particle sizes (*μ*m)	Solvent/ powder (v:w)	Yield (%)	TPC (mg GAE/g)	TFC (mg RUE/g)	TFnC (mg RUE/g)	TTC (mg TAE/g)
1	22	707	3	1.08	642.66	120.56	54.086	59.78
2	22	707	3	1.01	642.66	121.67	46.651	58.599
3	22	707	3	0.978	649.47	120.64	64.541	59.755
4	22	707	5	1.468	634.15	117.29	64.85	62.545
5	22	707	5	1.510	624.27	119.32	66.903	63.752
6	22	707	5	1.426	643.68	121.35	69.458	60.635
7	22	707	10	1.620	721.48	148.66	94.782	63.576
8	22	707	10	1.76	712.28	147.28	96.524	64.179
9	22	707	10	1.74	717.39	155.34	100.01	64.33
10	22	1400	5	1.468	634.15	117.29	64.85	62.545
11	22	1400	5	1.51	624.27	119.32	59.158	63.752
12	22	1400	5	1.426	643.68	121.35	69.458	60.635
13	22	707	5	2.96	713.14	137.39	68.451	62.362
14	22	707	5	2.94	695.96	127.56	81.036	63.046
15	22	707	5	2.8	704.38	127.09	69.884	62.191
16	22	250	5	3.04	727.95	135.05	71.82	62.621
17	22	250	5	3.08	700.7	139.11	73.95	60.61
18	22	250	5	2.88	692.87	137.56	72.904	60.032
19	22	707	5	1.468	634.15	117.29	64.85	62.545
20	22	707	5	1.51	624.27	119.32	59.158	63.752
21	22	707	5	1.426	643.68	121.35	69.458	60.635
22	55	707	5	0.652	679.95	129.44	67.406	56.864
23	55	707	5	0.676	673.04	123.62	64.618	54.879
24	55	707	5	0.652	671.56	120.88	65.509	55.507
25	70	707	5	1.09	663.07	87.771	42.624	52.667
26	70	707	5	1.1	647.18	91.921	48.626	50.756
27	70	707	5	0.98	642.88	87.094	40.146	49.7

GAE: gallic acid equivalent, RUE: rutin equivalent, TAE: tannic acid equivalent.

**Table 4:** Response surface central composite design and results for total antioxidant capacity (TAC), ferric ion reducing antioxidant power (FRAP), DPPH scavenging activity, and *α*-amylase and *α*-glucosidase inhibitory activities.

No.	Independent variables			Responses variables and their values				
	Extraction temperature (°C)	Powder particle sizes (*μ*m)	Solvent/powder (v:w)	TAC (mg AAE/g)	FRAP (mg AAE/g)	IC50 (DPPH-RSA)	IC50 (*α*-amylase inhibition)	IC50 (*α*-glucosidase inhibition)
1	22	707	3	39.705	144.45	34.896	61.15	37.133
2	22	707	3	40.318	142.36	34.624	69.745	35.298
3	22	707	3	39.854	152.22	34.774	62.555	39.714
4	22	707	5	39.151	157.18	34.558	43.7	32.977
5	22	707	5	38.957	159.99	36.522	49.315	32.967
6	22	707	5	39.391	154.37	36.086	43.815	32.321
7	22	707	10	37.88	157.35	32.54	42.182	21.106
8	22	707	10	40.542	160.76	37.984	47.222	21.088
9	22	707	10	40.453	170.35	35.85	47.92	20.35
10	22	1400	5	39.151	157.18	34.558	43.7	32.977
11	22	1400	5	38.957	159.99	36.522	49.315	32.967
12	22	1400	5	39.391	154.37	36.086	43.815	32.321
13	22	707	5	37.670	166.05	29.046	13.401	21.119
14	22	707	5	40.124	177.07	31.97	15.31	23.129
15	22	707	5	39.735	187.76	33.162	16.601	23.772
16	22	250	5	42.188	176.19	32.014	13.646	24.092
17	22	250	5	40.258	182.2	33.951	18.334	25.545
18	22	250	5	42.053	175.31	34.973	17.199	29.122
19	22	707	5	39.151	157.18	34.558	43.7	32.977
20	22	707	5	38.957	159.99	36.522	49.315	32.967
21	22	707	5	39.391	154.37	36.086	43.815	32.321
22	55	707	5	34.881	153.21	32.231	44.459	37.589
23	55	707	5	35.509	155.17	35.09	49.059	36.509
24	55	707	5	38.396	157.38	35.882	44.113	34.252
25	70	707	5	29.966	134.98	37.248	70.126	68.562
26	70	707	5	31.148	136.02	38.178	66.859	72.04
27	70	707	5	37.401	138.34	40.361	67.822	71.462

GAE: gallic acid equivalent, RUE: rutin equivalent, TAE: tannic acid equivalent, RSA: radical scavenging activities expressed as the inhibition percentage, IC_50_: concentrations (*µ*g/mL) of extracts that scavenged 50% of DPPH free radicals or inhibited 50% of enzyme activity.

**Table 5:** The second-order polynomial equation showing relationships between the response and factors studied.

Response variables	The second-order polynomial	*R*2	*R* ^2^ adjusted	*P* values
Extraction yield + 0.001362A^2^ + 0.000001B^2^ − 0.0727C^2^	Y = 2.93 − 0.1439A − 0.00366B + 1.043C	73.42	65.45	0.022
TPC + 0.000065B^2^ + 0.80C^2^	Y = 661.6 + 3.91A − 0.1709B − 0.1C − 0.0440A^2^	61.76	50.29	0.002
TFC − 0.05058A^2^ + 0.000022B^2^ + 0.627C^2^	Y = 90.7 + 3.941A − 0.0521B − 3.94C	93.45	91.49	0.000
TFnC − 0.02911A^2^ + 0.000004B^2^ − 0.113C^2^	Y = 8.0 + 2.170A − 0.0142B + 7.47C	89.91	86.88	0.000
TTC − 0.00235A^2^ − 0.000003B^2^ − 0.1678C^2^	Y = 51.47 − 0.020A + 0.00529B + 2.846C	93.93	92.11	0.000
TAC − 0.00292A^2^ + 0.000004B^2^ + 0.0694C^2^	Y = 44.98 + 0.137A − 0.00934B − 0.95C	75.52	68.18	0.000
FRAP − 0.0207A^2^ + 0.000019B^2^ − 1.272C^2^	Y = 106.8 + 1.34A − 0.0487B + 18.89C	72.92	64.79	0.000
DPPH-RSA (EC_50_) + 0.00575A^2^ + 0.000001B^2^ + 0.068C^2^	Y = 42.52 − 0.439A + 0.00080B − 0.79C	38.13	19.57	0.000
*α*-Amylase inhibition (EC_50_) + 0.0245A^2^ − 0.000023B^2^ + 2.370C^2^	Y = 132.7 − 1.57A + 0.0642B − 33.48C	74.77	67.20	0.000
*α*-Glucosidase inhibition (EC_50_) + 0.04377A^2^ − 0.000002B^2^ + 0.326C^2^	Y = 97.6 − 3.167A + 0.0086B − 6.60C	95.31	93.91	0.000

A: temperature (°C), B: powder particle size (*µ*m), and C: solvent-to-powder ratio (v:w).

**Table 6:** Predicted and actual experimental values of extraction yield, phenolic content, and antioxidant and antidiabetic activities under the optimal extraction conditions.

Responses	Predicted value	CI 95%	PI 95%	Experimental value
Yield (%)	3.055 ± 0.322	(2.383; 3.726)	(1.889; 4.220)	2.96 ± 0.08
TPC (mg GAE/g)	746.5 ± 17.0	(711.1; 782.0)	(684.9; 808.1)	717.05 ± 4.60
TFC (mg RUE/g)	155.63 ± 3.34	(148.68; 162.59)	(143.55; 167.72)	155.42 ± 1.80
TFnC (mg RUE/g)	94.59 ± 3.69	(86.88; 102.29)	(81.21; 107.96)	92.45 ± 3.01
TTC (mg TAE/g)	62.801 ± 0.816	(61.099; 64.502)	(59.846; 65.756)	61.07 ± 1.35
TAC (mg AAE/g)	41.41 ± 1.11	(39.10; 43.73)	(37.39; 45.44)	41.49 ± 1.07
FRAP (mg AAE/g)	185.31 ± 5.57	(173.68; 196.94)	(165.11; 205.51)	186.56 ± 1.47
EC_50_ (µg/mL) (DPPH-RSA)	33.62 ± 1.45	(30.60; 36.65)	(28.37; 38.88)	30.56 ± 2.74
EC_50_ (µg/mL) (*α*-amylase inhibition)	8.89 ± 7.08	(−5.89; 23.66)	(−16.77; 34.55)	15.05 ± 1.29
EC_50_ (µg/mL) (*α*- glucosidase inhibition)	16.03 ± 2.46	(10.89; 21.16)	(7.11; 24.94)	21.14 ± 0.77

CI: confidence interval; PI: prediction interval.

**Table 7:** Compounds identified by GC-MS analysis in *P. capitata*-optimized ethyl acetate extract.

No.	Retention index (iu)	Chemical markers	Peak areas (%)
1	609	Oxirane, propyl-	0.03
2	708	Oxirane, butyl-	0.012
3	907	Benzene, 1,3-dimethyl-	0.13
4	969	2,3,4,5-Tetrahydropyridazine	0.14
5	972	3-Methyl-5-methoxy-1-pentanol	0.30
6	1005	Octanal	2.10
7	1042	2H-pyran, tetrahydro-2-(2-propynyloxy)-	0.43
8	1068	1-Nonen-3-ol	0.37
9	1199	3-Undecene, 3-methyl-	0.02
10	1400	Tetradecane	7.35
11	1524	Benzene, 1-(1,5-dimethyl-4-hexenyl)-4-methyl-	53.14
12	1600	Hexadecane	3.21
13	1638	2-Butanone, 4-(4-hydroxy-3-methoxyphenyl)-	29.91
14	1916	3-Cyclopropyl carbonyl oxy tetradecane	0.02
15	1968	n-Hexadecanoic acid	0.54
16	943.4	R(-)3,7-Dimethyl-1,6-octadiene	0.15

iu: index unit.

**Table 8:** Effects of temperature variation on the chemical composition of the ethyl acetate extracts of *P. capitata* growing at the fruit bearing stage harvested on *Persea americana*.

Retention index (iu)	Chemical markers	Peak areas (%) in	
		Extract from 55°C	Extract from 70°C
769	Oxirane, propyl	2.39	98.72
2921	Methylbenzoyl-*β*-d- glucuronide triacetate	0.19	—
1204	Decanal	2.66	—
1638	Butan-2-one, 4-(3-hydroxy-2- methoxyphenyl)-	61.44	—
609	Oxirane, propyl-	0.91	—
1968	n-Hexadecanoic acid	18.42	—
1708	4-Hydroxy-3-methoxyphenylglycol	12.98	—

## Data Availability

The data of this manuscript are available on request from the corresponding author.

## Abbreviations

CAM: Cameroon
DMSO: Dimethyl sulfoxide
DPPH: 2,2-Diphenyl-1-picrylhydrazyl
FRAP: Ferric ion reducing antioxidant power
GAE: Gallic acid equivalent
GC-MS: Gas chromatography-mass spectrometry
HNC: Cameroon National Herbarium
IC_50_: Concentrations (*µ*g/mL) of extracts that scavenge 50% of DPPH free radicals or inhibit 50% of enzyme activity
iu: Index unit
NIST: National Institute of Standards and Technology
RAS: Radical scavenging activity
RSD: Response surface design-type central composite
GC: Gas chromaoyography
RUE: Rutin equivalent
SRF: Cameroon forest reserve society
TAC: Total antioxidant capacity
TAE: Tannic acid equivalent
TFC: Total flavonoid content
TFnC: Total flavonol content
TPC: Total phenolic content
TTC: Total tannin content
v:w: Volume/weight.

## References

[1] WHO (2018). *Traditional and Complementary Medicine in Primary Health Care*.

[2] Oyebode O., Kandala N. B., Chilton P. J., Lilford R. J. (2016). Use of traditional medicine in middle-income countries: a WHO-SAGE study. *Health Policy and Planning*.

[3] Mirzaeian R., Sadoughi F., Tahmasebian S., Mojahedi M. (2019). Progresses and challenges in the traditional medicine information system: a systematic review. *Journal of Pharmacy Pharmacognosy Research*.

[4] Sachan A. K., Vishnoi G., Kumar R. (2016). Need of standardization of herbal medicines in modern era. *International Journal of Phytomedicine*.

[5] Chirag P., Tyagi S., Kanu J. P., Tushur P., Harnish K. P., Priyanka H. P. (2014). Standardization of herbal medecine: a concise review. *Journal of Pharmacy and Biology Research*.

[6] Abu Bakar F. I., Abu Bakar M. F., Abdullah N., Endrini S., Fatmawati S. (2020). Optimization of extraction conditions of phytochemical compounds and anti-gout activity of *Euphorbia hirta* L. (Ara tanah) using response surface methodology and liquid chromatography-mass spectrometry (LC-MS) analysis. *Evidence-based Complementary and Alternative Medicine*.

[7] Rezazi S., Abdelmalek S., Hanini S. (2017). Kinetic study and optimization of extraction process conditions. *Energy Procedia*.

[8] Koffi A. A., Kouassi F., N’Goran S. B. K., Soro D. (2015). Les Loranthaceae, parasites des arbres et arbustes: cas du département de Katiola, au nord de la Côte d’Ivoire. *International Journal of Brain and Cognitive Sciences*.

[9] Ohikhena F. U., Wintola O. A., Afolayan A. J. (2016). Toxicity assessment of different solvent extracts of the medicinal plant, Phragmanthera capitata (Sprengel) Balle on brine shrimp (Artemia salinaArtemia salina). *International Journal of Pharmacology*.

[10] Amon A. D., Seguena F., Soro K., N’guessan K. (2017). Ethnobotany study of Loranthaceae, hemiparasitic plants used in traditional medicine by population, in the Sud-Comoé region (Côte d’Ivoire). *Journal of Medical Plants and Studies*.

[11] Ladoh-Yemeda C. F., Ndongo D., Tomedi E. M. (2019). Medicinal potentials of Phragmanthera capitata (Sprengel) S. Balle used in the city of Douala (Cameroon). *Haya Saudi Journal of Life Science*.

[12] Feudjio C., Njateng G. S. S., Kuiate J. (2018). Evaluation of antidiabetic activity of 518 aqueous extract of leaves from Phragmanthera capitata (Sprengel) S. Balle 519 (Laurenthaceae) in Wistar albino Rats. *Journal of Diseases and Medicinal Plants*.

[13] Obi B. C., Igweze Z., Nwaogu V., Ben C., Akunne T. C. (2019). Studies on the anticonvulsant and sedative effects of Jatropha curcas (Euphorbiaceae) and Phragmanthera capitata (Loranthaceae) in mice. *Drug Discovery*.

[14] Takem L. P., Poh C. F., Kechi E. L., Abe N. P. (2014). Steroidogenetic and spermatogenetic activities of aqueous extract of Phragmanthera capitata in Wistar rats. *International Journal of Pharmacy and Science*.

[15] Takem L. P., Essien A. D., Udia P. M., Anele E. I. (2016). Evaluation of lipogenic property of Phragmanthera capitata in diabetic rats. *The Journal of Phytopharmacology*.

[16] Ohikhena F. U., Wintola O. A., Afolayan A. J. (2017). Evaluation of the antibacterial and antifungal properties of Phragmanthera capitata (sprengel) balle (Loranthaceae), a mistletoe growing on rubber tree, using the dilution techniques. *Science World Journal*.

[17] Etame-Loe G., Okalla Ebongue C., Ngaba G. P. (2018). Évaluation des activités antioxydante et anti-inflammatoire de l’extrait aqueux de l’haustorium de Phragmanthera capitata (Sprengel) S. Balle (Loranthaceae) récolté sur Psidium guajava sur les rats femelles adultes de la souche wistar. *Journal of Animal and Plant Science*.

[18] Ohikhena F. U., Wintola O. A., Afolayan A. J. (2018). Investigating the antidiabetic potential of Phragmanthera capitata, a mistletoe harvested from rubber tree. *Journal of Herbs, Spices, & Medicinal Plants*.

[19] Feudjio C., Yameen M. A., Singor Njateng G. S. (2020). The influence of solvent, host, and phenological stage on the yield, chemical composition, and antidiabetic and antioxidant properties of Phragmanthera capitata (Sprengel) S. Balle, Evidence-based Complement. *Alternative Medicine*.

[20] Ohikhena F. U., Wintola O. A., Afolayan A. J. (2018). Quantitative phytochemical constituents and antioxidant activities of the mistletoe, Phragmanthera capitata (Sprengel) Balle extracted with different solvents. *Pharmacognosy Research*.

[21] Laib I., Barkat M. (2018). Optimization of conditions for extraction of polyphenols and the determination of the impact of cooking on total polyphenolic, antioxidant, and anticholinesterase activities of potato. *Foods*.

[22] Edirs S., Turak A., Numonov S., Xin X., Aisa H. A. (2017). Optimization of extraction process for antidiabetic and antioxidant activities of Kursi Wufarikun Ziyabit using response surface methodology and quantitative analysis of main components. *Evidence-based Complementary and Alternative Medicine*.

[23] Antony A., Farid M. (2022). Effect of temperatures on polyphenols during extraction. *Applied Sciences*.

[24] Sulaiman S. I. C., Basri M., Masoumi H. R. F., Chee W. J., Ashari S. E., Ismail M. (2017). E ects of temperature, time, and solvent ratio on the extraction of phenolic compounds and the anti-radical activity of Clinacanthus nutans Lindau leaves by response surface methodology. *Chemistry Central Journal*.

[25] Radojkovi M., Zekovi Z., Joki S., Vidovi S., Milo S. (2012). Optimization of solid-liquid extraction of antioxidants from black mulberry leaves by response surface methodology. *Food Technology and Biotechnology*.

[26] Azahar N. F., Gani S. S. A., Mohd Mokhtar N. F. (2017). Optimization of phenolics and flavonoids extraction conditions of Curcuma zedoaria leaves using response surface methodology. *Chemistry Central Journal*.

[27] Frempong T. F., Boadi N. O., Badu M. (2021). Optimization of extraction conditions for polyphenols from the stem bark of Funtumia elastica (Funtum) utilizing response surface methodology. *AAS Open Res*.

[28] Mokrani A., Madani K. (2016). Effect of solvent, time and temperature on the extraction of phenolic compounds and antioxidant capacity of peach (Prunus persica L.) fruit. *Separation and Purification Technology*.

[29] Roselló-Soto E., Marti-Quijal F., Cilla A. (2019). Influence of temperature, solvent and pH on the selective extraction of phenolic compounds from Tiger Nuts by-products: triple-TOF-LC-MS-MS characterization. *Molecules*.

[30] Altemimi A., Watson D. G., Kinsel M., Lightfoot D. A. (2015). Simultaneous extraction, optimization, and analysis of flavonoids and polyphenols from peach and pumpkin extracts using a TLC-densitometric method. *Chemistry Central Journal*.

[31] Baharuddin N. S., Roslan M. A. M., Bawzer M. A. M. (2021). Response surface optimization of extraction conditions and in vitro antioxidant and antidiabetic evaluation of an under-valued medicinal weed, mimosa pudica. *Plants*.

[32] Bilal A., Rehman M. U., Amin I. (2015). A review on pharmacological properties of zingerone (4-(4-Hydroxy-3-methoxyphenyl)-2-butanone). *Science World Journal*.

[33] Oyebode O. A., Erukainure O. L., Koorbanally N. A., Islam M. S. (2018). Acalypha wilkesiana ‘java white’: identification of some bioactive compounds by gc-ms and their effects on key enzymes linked to type 2 diabete. *Acta Pharmaceutica*.

[34] Rusmana D., Wahyudianingsih R., Elisabeth M., Balqis B., Maesaroh M., Widowati W. (2017). Antioxidant activity of Phyllanthus niruri extract, rutin and quercetin. *Indonesia Biomedical Journal*.

[35] Medini F., Fellah H., Ksouri R., Abdelly C. (2014). Total phenolic, flavonoid and tannin contents and antioxidant and antimicrobial activities of organic extracts of shoots of the plant Limonium delicatulum. *Journal of Taibah University for Science*.

[36] Sales P. M., Souza P. M., Simeoni L. A., Magalhães P. O., Silveira D. (2012). *α*-amylase inhibitors: a review of raw material and isolated compounds from plant source. *Journal of Pharmacy & Pharmaceutical Sciences*.

[37] Patrautanu O. A., Lazăr L., Popa V. I., Volf I. (2019). Influence of particle size and size distribution on kinetic mechanism of spruce bark polyphenols extraction. *Cellulose Chemistry and Technology*.

[38] Vuong Q. V., Golding J. B., Stathopoulos C. E., Nguyen M. H., Roach P. D. (2011). Optimizing conditions for the extraction of catechins from green tea using hot water. *Journal of Separation Science*.

[39] Sheng Z., Zhao J., Muhammad I., Zhang Y. (2018). Optimization of total phenolic content from Terminalia chebula Retz. fruits using response surface methodology and evaluation of their antioxidant activities. *PLoS One*.

[40] Makanjuola S. A. (2017). Influence of particle size and extraction solvent on antioxidant properties of extracts of tea, ginger, and tea-ginger blend. *Food Sciences and Nutrition*.

[41] Wang Y., Wang C., Xue H., Jin Y., Yang M., Leng F. (2022). Comparative analysis of three kinds of extraction kinetic models of crude polysaccharides from codonopsis pilosula and evaluate the characteristics of crude polysaccharides. *Biomass Convers and Biorefinery*.

[42] Zaiter A., Becker L., Karam M. C., Dicko A. (2016). Effect of particle size on antioxidant activity and catechin content of green tea powders. *Journal of Food Science & Technology*.

[43] Tchabo W., Ma Y., Kwaw E., Xiao L., Wu M., T Apaliya M. (2018). Impact of extraction parameters and their optimization on the nutraceuticals and antioxidant properties of aqueous extract mulberry leaf. *International Journal of Food Properties*.

[44] Sulaiman K. S., Mustafa M., Hafiz I., Nulit R., Rusea G. (2014). Effect of drying methods, solid-solvent ratio, extraction time and extraction temperature on phenolic antioxidants and antioxidant activity of Guiera senegalensis J.F. Gmel (Combretaceae) leaves water extract. *American Journal of Phytomedicine and Clinical Therapeutics*.

[45] Nasma A., Aishath N., Azilah A., Sulaiman A. Z. (2018). Optimization of vitexin and isovitexin compounds extracted from dried Mas Cotek leaves using one-factor-at-a-time (OFAT) approach in aqueous extraction. *International Food Research Journal*.

[46] Predescu N. C. (2016). The influence of solid-to-solvent ratio and extraction method on total phenolic content, flavonoid content and antioxidant properties of some ethanolic plant extracts. *Revue Chimique*.

[47] Andres A. I., Petron M. J., Lopez A. M., Timon M. L. (2020). Optimization of extraction conditions to improve phenolic content and in vitro antioxidant activity in Craft Brewers’ spent grain using response surface methodology (RSM). *Foods*.

[48] Montenegro-Landívar M. F., Tapia-Quiros P., Vecino X. (2021). Recovery of added-value compounds from orange and spinach processing residues: green extraction of phenolic compounds and evaluation of antioxidant activity. *Antioxidants*.

[49] Clodoveo M. L., Crupi P., Muraglia M., Corbo F. (2022). Ultrasound assisted extraction of polyphenols from Ripe Carob Pods (Ceratonia siliqua L.): combined designs for screening and optimizing the processing parameters. *Foods*.

[50] Venkataramanamma D., Aruna P., Singh R. P. (2016). Standardization of the conditions for extraction of polyphenols from pomegranate peel. *Journal of Food Science & Technology*.

